# Effects of prescription restrictive interventions on antibiotic procurement in primary care settings: a controlled interrupted time series study in China

**DOI:** 10.1186/s12962-018-0086-y

**Published:** 2018-01-16

**Authors:** Yuqing Tang, Chaojie Liu, Zinan Zhang, Xinping Zhang

**Affiliations:** 1 School of Medicine and Health Management, Tongji Medical College, Huazhong University of Science and Technology, Wuhan, 430030 People’s Republic of China; 2 School of Psychology and Public Health, La Trobe University, Kingsbury Drive, Melbourne, VIC 3086 Australia

**Keywords:** Administrative regulation, Restrictive intervention, Antibiotics, Primary care, China

## Abstract

**Background:**

The overuse of antibiotics has been identified as a major challenge in regard to the rational prescription of medicines in low and middle income countries. Extensive studies on the effectiveness of persuasive interventions, such as guidelines have been undertaken. There is a dearth of research pertaining to the effects of restrictive interventions. This study aimed to evaluate the impacts of prescription restrictions in relation to types and administration routes of antibiotics on antibiotic procurement in primary care settings in China.

**Methods:**

Data were drawn from the monthly procurement records of medicines for primary care institutions in Hubei province over a 31-month period from May 2011 to November 2013. We analyzed the monthly procurement volume and costs of antibiotics. Interrupted time series analyses with a difference-in-difference approach were performed to evaluate the effect of the restrictive intervention (started in August 2012) on antibiotic procurement in comparison with those for cardiovascular conditions. Sensitivity tests were performed by replacing outliers using a simple linear interpolation technique.

**Results:**

Over the entire study period, antibiotics accounted for 33.65% of the total costs of medicines procured for primary care institutions: mostly non-restricted antibiotics (86.03%) and antibiotics administered through parenteral routes (79.59%). On average, 17.14 million defined daily doses (DDDs) of antibiotics were procured per month, with the majority (93.09%) for non-restricted antibiotics and over half (52.38%) for parenteral administered antibiotics. The restrictive intervention was associated with a decline in the secular trend of costs for non-restricted oral antibiotics (− 0.36 million Yuan per month, p = 0.029), and for parenteral administered restricted antibiotics (− 0.28 million Yuan per month, p = 0.019), as well as a decline in the secular trend of procurement volume for parenteral administered non-restricted antibiotics (− 0.038 million DDDs per month, p = 0.05).

**Conclusions:**

Restrictive interventions are effective in reducing the procurement of antibiotics. However, the effect size is relatively small and antibiotic consumptions remain high, especially parenteral administered antibiotics.

## Background

The overuse of antibiotics has been identified as a major global challenge, especially in low and middle income countries (LMIC) [1, 2]. It has been proved to be associated with the development and spread of antimicrobial resistance (AMR). Rising AMR levels, in combination with a lack of new effective antibiotics, increases the morbidity and mortality of infectious diseases [3]. The overuse of antibiotics also drives inflation related to healthcare costs.

Empirical evidence shows that irrational antibiotic prescriptions are most prevalent in primary care settings [4]. The overuse of antibiotics for upper respiratory tract infections in primary care, for instance, was observed worldwide [5]. Antibiotic abuse has also been identified as a serious problem in China [6]. The primary health network in China includes community/township health centres and outreach stations/clinics. They provide essential medical care services (covering outpatient, inpatient and rehabilitation care in line with the Essential Medicines List policy) and essential public health services (including personal health records, health education, planned immunization, child (0–6 years) health care, maternity care, aged care, management of chronic conditions (hypertension and diabetes), management of severe mental disorders, management of tuberculosis, use of Chinese medicines for health promotion, reporting and emergency response to infectious diseases, supporting health inspection activities, free supply of contraception, and improving the health literacy of consumers) [7]. A growing body of literature has revealed a very high level of use of antibiotics in primary care settings in China [8]. The direct cost associated with the overuse of antibiotics in China is estimated to be around 2.91–13.93 billion yuan ($0.42–2.02 billion USD) per year [9]. A recent national survey shows that 52.9% of patients visiting primary care institutions in China were prescribed antibiotics, but only 39.4% of those who received antibiotics needed them based on their clinical condition [10].

Increased AMR triggered a surge of interventions on antibiotic prescribing practices [11]. Clinical guidelines are perhaps the most commonly used instrument for promoting rational prescriptions. Guidelines alone may play a limited role in changing prescribing practices [12, 13]. In a systematic review, Ivers and colleagues found that audit and feedback can bring about 70% compliance with prescription guidelines, leading to a 16% reduction in antibiotic prescriptions [14]. Nonetheless, the current intervention strategies have been heavily biased towards persuasive measures (such as guidelines), and restrictive interventions (such as administrative rules on prescribers) are rare both in practice and in research [15]. Some researchers argue that restrictive interventions may have great potential for curbing antibiotic abuse [14, 16].

In China, both persuasive and restrictive measures have been used to address antibiotic over-prescriptions [17]. In the recent round of health system reform launched in 2009, access to antibiotics in primary care facilities has been restricted to medicines listed in the Essential Medicines List (EML) and these medicines have to be sold with zero-markup [18]. Unfortunately, limited effects on antibiotic prescribing practices have been observed after a few years of implementing these policies [19–21]. In 2012, the Chinese government issued ‘‘administrative rules for the clinical use of antibiotics’’, which are considered the most rigid regulatory control over antibiotic prescriptions to date [22, 23].

This study aimed to evaluate the effects of the “administrative rules” on antibiotic prescriptions in primary care in Hubei province. Hubei’s “administrative rules for clinical use of antibiotics” involve three major components [24]: (1) antibiotics are categorized into three groups—non-restricted, restricted and controlled. The EML for primary care contains non-restricted and restricted antibiotics only; (2) administrative restrictions are imposed on health facilities and medical practitioners in relation to prescriptions of restricted and controlled antibiotics, as well as intravenous infusion of antibiotics; (3) penalties apply to those who violate the rules (Box 1).

### Box 1 Administrative rules for the clinical use of antibiotics

#### 1. Antibiotic categorization

Antibiotics are categorized into three groups: non-restricted, restricted, and controlled. Non-restricted antibiotics can be used for the prevention and treatment of mild infections. Restricted antibiotics can be used for severe infections, infections in patients with immune dysfunction, and infected pathogens that are sensitive only to restricted antibiotics. Controlled antibiotics can only be used in special circumstances. Detailed guidelines were issued by the government in relation to the type of antibiotics that applies to various clinical conditions and evidence that is required to justify antibiotic prescriptions.

#### 2. Prescribing authorization

Prescribing privileges are conditional to qualification, professional title, and training of prescribers. Only doctors with a middle or senior professional title are allowed to prescribe controlled antibiotics. Medical practitioners without a professional title (such as assistant doctors) are not allowed to prescribe restricted antibiotics. Health workers in village clinics can only prescribe non-restricted antibiotics. The intravenous infusion of antibiotics in village/community clinics is subject to approval from county health bureaux.

#### 3. Pharmaceutical management committee

Secondary and tertiary hospitals are required to establish a pharmaceutical management committee, consisting of representatives of physicians, pharmacists, microbiologists, and managers. Primary care institutions are required to establish an antimicrobial working group.

#### 4. Monitoring and evaluation of antibiotic prescriptions

Antibiotic prescriptions should be audited on a regular basis in line with the prescribing guidelines.

#### 5. Penalty

Institutions that violate the rules are subject to penalties imposed by the health authorities, which include downgraded accreditation and dismissal of managers. Medical practitioners involved may lose permission to prescribe antibiotics, have their medical registration revoked, or prosecuted.

## Methods

### Setting

This study was conducted in Hubei province. Hubei is located in central China, with a population of over 61 million across a geographic area of 185,900 km^2^. The annual average income per capita in Hubei ranks in the middle range of all provinces: 6898 yuan ($1000 USD) for rural residents and 18,374 yuan ($2659 USD) for urban residents (2012).

Data used in this study were extracted from the procurement database for urban community health centers and rural township health centers. There are 1430 community/township health centers in Hubei. The procurement of medicines for the 1235 state-owned community/township health centers is made through a provincial tendering system managed by the Hubei Medical Procurement Administrative Agency (HMPA). Primary care institutions are only allowed to stock and dispense medicines listed in the EML at zero markup. The procurement system covers all medicines listed in the EML, including 32 generic non-restricted antibiotics and 4 generic restricted antibiotics.

### Study design

The procurement system recorded volumes and prices of medicines delivered to community/township health centers. The medicines were coded using the Anatomical Therapeutic Chemical (ATC) coding system. We compared the changes in volumes and costs of antibiotics for systemic use (ATC code, J01) with those of medicines used for the cardiovascular system (ATC code, C).

We adopted a controlled interrupted time series design with a difference-in-difference approach. An interrupted time series is a strong quasi-experimental design in which data are collected at multiple time points before and after the intervention. The advantage of this design is that it can detect a possible underlying secular trend which occurs after the intervention. By adding a control group, it is possible to separate the intervention effect from other confounding effects that may have occurred at the same time [25–27]. In this study, the ATC “C” group of medicines served as a control group because it contained large volumes of orders and it is not subject to the influence of the restrictive intervention tested in this study.

The design of this study was further strengthened by adopting a difference-in-difference approach, which enables us to estimate the effect size of the restrictive intervention, adjusting for pre-existing differences and the confounding influence of other factors.

### Data collection and management

Data were extracted from the HMPA procurement system, which contained monthly records in relation to the unit strength, pack size, price, procurement volume, and total cost of each delivered medicine. Procured medicines in the ATC “J01” group included 32 products classified as non-restricted antibiotics and 4 products classified as restricted antibiotics, compared with 39 products in the ATC “C” group.

The procurement records over a 31-month period (from May 2011 to November 2013) were collected. The administrative restrictive rules on antibiotic prescriptions were introduced in August 2012. This resulted in a final sample of 15 months of pre-intervention records and 16 months of post-intervention records. We discontinued the data collection in December 2013 because a new version of EML was introduced at that time.

### Statistical analysis

Two indicators were used to examine the outcomes of the restrictive intervention: volume and costs of procured medicines. The cost of the procured medicines was calculated based on the price and volume of each product, without adjustment for present values. The volume of procured medicines was measured using defined daily dose (DDD, the average maintenance dose per day for a drug used for its main indication in adults), a measurement developed by the World Health Organization (WHO) to compare drug consumptions. According to the WHO Collaborative Centre for Drug Statistics Methodology [28], DDD equivalence per package (DPP) of medicines was calculated in DDD units [DPP = (unit strength × pack size/DDD)]. The total volume for each group of procured medicines (DDDs) was estimated as the summed DPPs of all-inclusive products [29].

$$ DDDs = \sum\limits_{i = 1}^{n} {\left( {DPP_{i} \times N_{i} } \right)} $$

*N*_*i*_ represents the number of packages of certain product (*i*) delivered to the community/township health centers.

To estimate the effect of the intervention on the outcome variables, the following segmented linear regression model was developed [25]:

$$ {Y}_{t} = \,\beta_{0} + \,\beta_{1} \cdot {T}_{t} +\beta_{2} \cdot {I}_{t} +\beta_{3} \cdot {T \;{\text{after}}\; I}_{t} +\beta_{4} \cdot {G} +\beta_{5} \cdot {G} \cdot {T}_{t} +\beta_{6} \cdot {G} \cdot {I}_{t} +\beta_{7} \cdot {G} \cdot {T \;{\text{after}}\; I}_{t} +\beta_{8} \cdot \sin (2\pi{T}_{t} /12) + \,\beta_{9} \cdot \cos (2\pi{T}_{t} /12)\varepsilon_{t} $$

In this model, *Y*_*t*_ is the outcome indicator in month *t*; *T*_*t*_ is a continuous variable indicating the months passed at month *t* since the start of the observation period; *I*_*t*_ represents the two periods before (value = 0) and after (value = 1) the intervention; *T* is a continuous variable indicating months passed since the intervention (time prior to the intervention is coded 0); *G* represents the two groups (0 for the control group and 1 for antibiotic group); *β*_6_ estimates the mean difference in pre-post (intervention) changes between the two groups in relation to the outcome indicators (the effect size of the intervention); *β*_7_ estimates the difference in secular trend changes in time series between the two groups (the change in trend due to the intervention). *β*_8_ and *β*_9_ were used to correct for a potential seasonality effect [30]. To correct for dependency of time series data, New-West standard errors were calculated in these models [31].

To better understand the changing pattern of prescribing practices, we also estimated the effects of the intervention on the consumption of non-restricted antibiotics, restricted antibiotics, oral antibiotics, and antibiotics administered through the parenteral route, respectively.

We observed two obvious outliners (at time point 21 and 22) which were caused by the holiday season (Chinese New Year). Sensitivity tests were performed by replacing the outliers using a simple linear interpolation technique [32].

All of the analyses were performed using Stata 15.0 (Stata Corp LP, College Station, TX, USA).

## Results

**Fig. 1:**
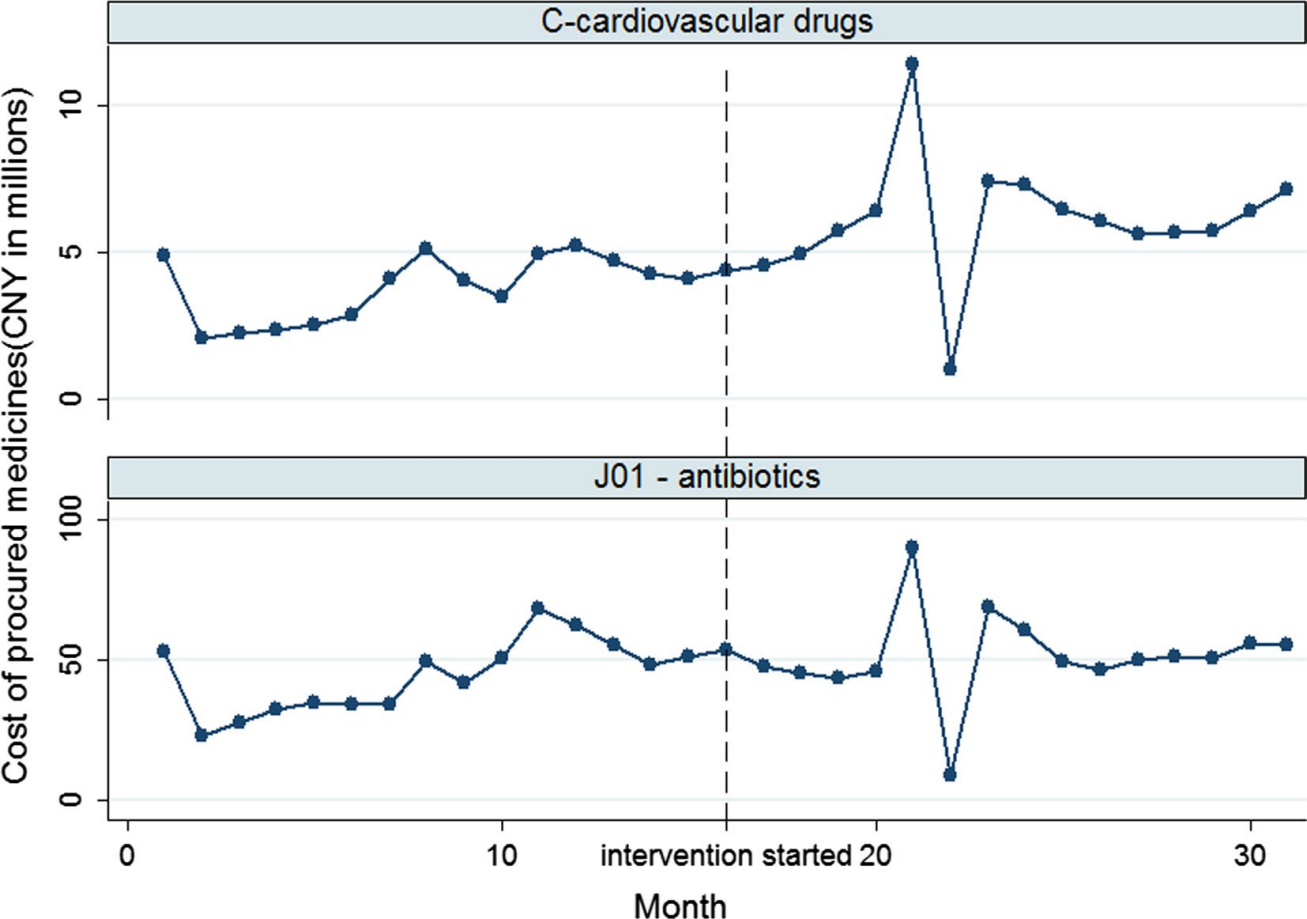
Time trend of monthly costs for procured medicines: antibiotics vs medicines for cardiovascular system

Overall, antibiotics accounted for 33.65% of the total procurement costs for all drugs (36.10% before intervention and 32.11% after intervention). On average, ¥47.97 million yuan per month were spent on antibiotics (¥44.31 million per month before intervention and ¥51.41 million after intervention). The percentage increase in costs over time was smaller for antibiotics than for cardiovascular medicines (Fig. 1).

**Fig. 2:**
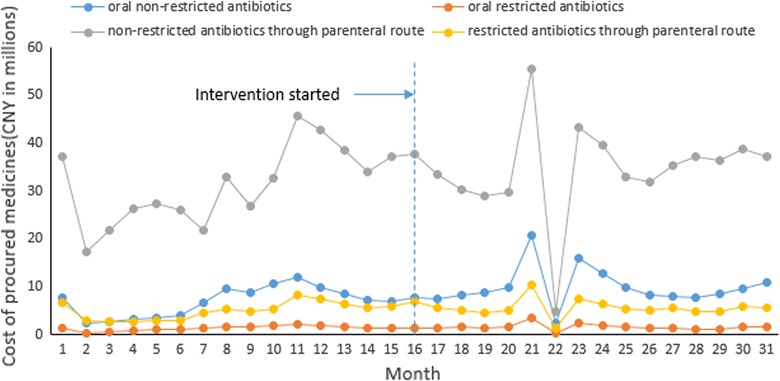
Time trend of monthly costs for antibiotic subgroup

Non-restricted antibiotics accounted for 86.03% of the total cost of antibiotics (17.43% oral and 68.6% parenteral), while restricted antibiotics accounted for 13.97% of the total cost of antibiotics (2.98% oral and 10.99% parenteral). As a group, parenteral antibiotics accounted for almost 80% (79.59%) of the total cost of antibiotics (Fig. 2).

**Fig. 3:**
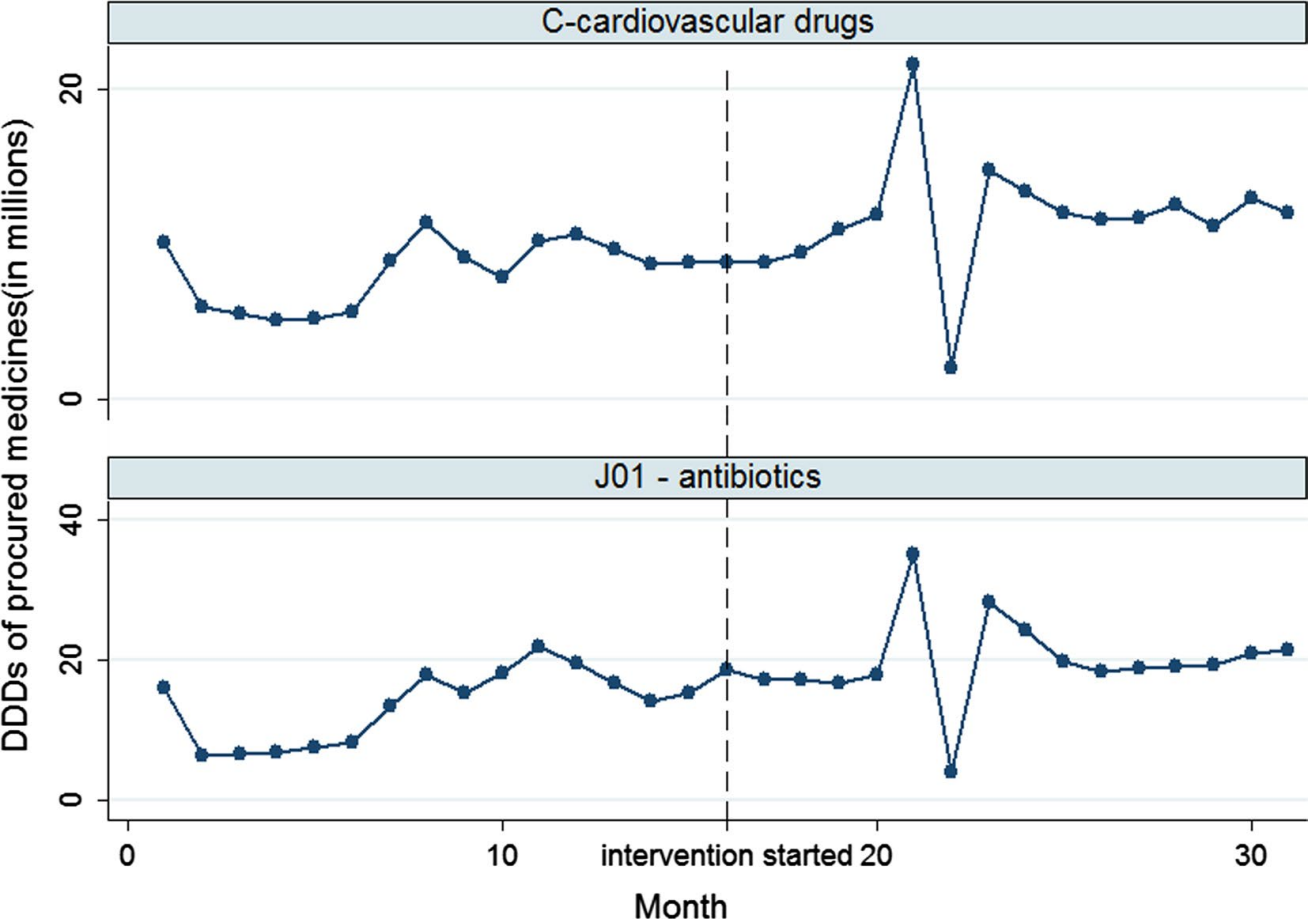
Time trend of monthly DDDs for procured medicines: antibiotics vs medicines for the cardiovascular system

On average, 16.79 million DDDs of antibiotics were procured per month (13.60 million before intervention and 19.78 million after intervention), compared with 10.01 million DDDs of medicines for the cardiovascular system (8.24 million before intervention and 11.66 million after intervention). The percentage increase in DDDs over time was smaller for antibiotics than for cardiovascular medicines (Fig. 3).

**Fig. 4:**
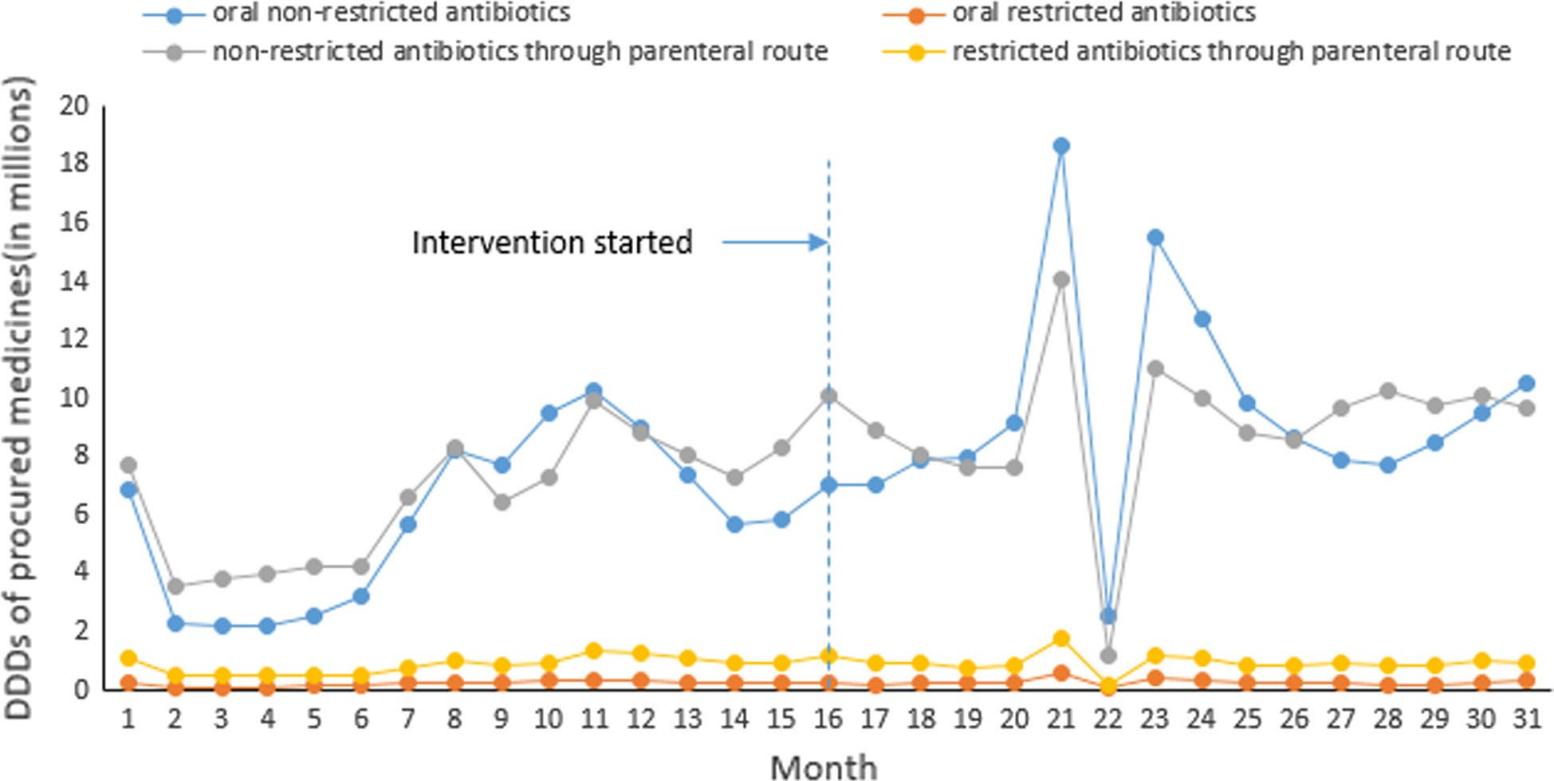
Time trend of monthly antibiotic DDDs by subgroups

Non-restricted antibiotics accounted for 93.09% of the DDDs for antibiotics (46.11% oral and 46.98% parenteral), while restricted antibiotics accounted for 6.91% of the total cost of antibiotics (1.52% oral and 5.39% parenteral). As a group, parenteral antibiotics accounted for more than half (52.38%) of the DDDs for antibiotics (Fig. 4), despite their much higher contribution to the cost of antibiotics.

The segmented linear regression models revealed that the intervention was associated with a decline in the secular trend of costs for non-restricted oral antibiotics (− 0.36 million Yuan per month, p = 0.029) compared to the control. Prior to the intervention, there was an increasing secular trend (average increase per month 0.25 million Yuan, p = 0.021). The intervention was associated with a 27.84% reduction in the cost of non-restricted oral antibiotics. The intervention was also associated with a decline in the secular trend of costs for restricted antibiotics administered through the parenteral route (− 0.28 million Yuan per month, p = 0.019) compared to the control. Prior to the intervention, there was not a significant increasing secular trend (p = 0.114). The interventions were associated with a 33.64% reduction in the cost of restricted antibiotics administered through the parenteral route. No significant trend changes were observed for the costs of non-restricted antibiotics administered through the parenteral route or oral restricted antibiotics. Despite the secular trend changes, the mean differences in the magnitude of changes remained statistically insignificant (Table 1).

**Table 1:** Effects of the intervention on the cost of antibiotics in comparison with controls: findings from the segmented linear regression models

Outcome indicators	Original models		Models with replaced outliers	
	Coefficient [95% CI]	p	Coefficient [95% CI]	p
Total cost for antibiotics (vs controls)				
Mean difference in pre-post changes (million yuan)	− 9.02 [− 26.74, 8.69]	0.312	− 8.31 [− 18.68, 2.06]	0.114
Difference in secular trend changes (million yuan per month)	− 1.58 [− 3.20, 0.03]	0.054	− *1.56 [*− *2.96,* − *0.15]*	*0.030*
Cost for non-restricted antibiotics (vs controls)				
Oral antibiotics (vs controls)				
Mean difference in pre-post changes (million yuan)	− 0.80 [− 5.05, 3.44]	0.706	− 0.76 [− 3.56, 2.03]	0.585
Difference in secular trend changes (million yuan per month)	− *0.36 [*− *0.68, *− *0.04]*	*0.029*	− *0.35 [*− *0.61,* − *0.079]*	*0.012*
Antibiotics administered through parenteral route (vs controls)				
Mean difference in pre-post changes (million yuan)	− 7.35 [− 18.71, 4.00]	0.200	− 6.61 [− 13.64, 0.42]	0.065
Difference in secular trend changes (million yuan per month)	− 0.85 [− 1.93, 0.22]	0.118	− 0.85 [− 1.80, 0.11]	0.080
Cost for restricted antibiotics (vs controls)				
Oral antibiotics (vs controls)				
Mean difference in pre-post changes (million yuan)	− 0.74 [− 2.74, 1.26]	0.463	− 0.77 [− 1.55, 0.01]	0.053
Difference in secular trend changes (million yuan per month)	− 0.032 [− .19, 0.121]	0.675	− 0.036 [− 0.15, 0.08]	0.529
Antibiotics administered through parenteral route (vs controls)				
Mean difference in pre-post changes (million yuan)	− 0.63 [− 0.29, 1.63]	0.577	− 0.69 [− 2.28, 0.90]	0.388
Difference in secular trend changes (million yuan per month)	− *0.28 [*− *0.52 ,* − *0.05]*	*0.019*	− *0.27 [*− *0.49,* − *0.06]*	*0.015*

Italic values indicate significance of p value (p < 0.05)

The intervention was associated with a decline in the secular trend of the procurement volume of restricted antibiotics administered through the parenteral route (− 0.038 million DDDs per month, p = 0.05) compared to the control. Prior to the intervention, there was not a significant increasing secular trend (p = 0.128). The interventions were associated with a 26.82% reduction in the volume of restricted antibiotics administered through the parenteral route. No significant trend changes were observed for the volumes of non-restricted antibiotics, oral restricted antibiotics, and oral restricted antibiotics administered through the parenteral route. Again, the mean differences in the magnitude of volume changes remained statistically insignificant (Table 2).

**Table 2:** Effects of the intervention on procurement volumes of antibiotics in comparison with controls: findings from the segmented linear regression models

Outcome indicators	Original models		Models with replaced outliers	
	Coefficient [95% CI]	p	Coefficient [95% CI]	p
Total DDDs for antibiotics (vs controls)				
Mean difference in pre-post changes (DDDs in millions)	− 0.59 [− 7.96, 6.79]	0.874	− 0.32 [− 3.92, 3.27]	0.857
Difference in secular trend changes (DDDs in millions per month)	− 0.51 [− 1.09, 0.080]	0.089	*−* *0.51 [−* *0.97, −* *0.05]*	*0.031*
DDDs for non-restricted antibiotics (vs controls)				
Oral antibiotics (vs controls)				
Mean difference in pre-post changes (DDDs in millions)	0.11 [− 4.74, 4.96]	0.964	0.18 [*−* 1.77, 2.14]	0.852
Difference in secular trend changes (DDDs in millions per month)	− 0.22 [− 0.56, 0.13]	0.213	*−* 0.22 [*−* 0.47,0.02]	0.073
Antibiotics administered through parenteral route (vs controls)				
Mean difference in pre-post changes (DDDs in millions)	*−* 0.40 [*−* 4.27, 3.47]	0.837	*−* 1.43 [*−* 4.60, 1.74]	0.370
Difference in secular trend changes (DDDs in millions per month)	0.03 [*−* 0.27, 0.33]	0.849	0.08 [*−* 0.21, 0.37]	0.595
DDDs for restricted antibiotics (vs controls)				
Oral antibiotics (vs controls)				
Mean difference in pre-post changes (DDDs in millions)	*−* 0.24 [*−* 4.06, 3.58]	0.899	*−* 0.33 [*−* 2.08, 1.42]	0.704
Difference in secular trend changes (DDDs in millions per month)	0.066 [*−* 0.23, 0.37]	0.660	0.055 [*−* 0.18, 0.29]	0.642
Antibiotics administered through parenteral route (vs controls)				
Mean difference in pre-post changes (DDDs in millions)	*−* 0.16 [*−* .54, 0.229]	0.420	*−* 0.18 [*−* 0.44, 0.086]	0.183
Difference in secular trend changes (DDDs in millions per month)	*−* *0.038 [−* *0.075, 0.00]*	*0.050*	*−* *0.035 [−* *0.069, 0.001]*	*0.043*

Italic values indicate significance of p value (p < 0.05)

The results of the regression models using data with and without replacing outliers were consistent, with similar coefficients for the change in the secular trend of antibiotic prescriptions: − 1.58 vs − 1.56 for total cost; − 0.51 vs − 0.51 for total DDDs.

## Discussion

The restrictive intervention on antibiotic prescriptions is associated with some positive changes. This study demonstrated that the restrictive intervention resulted in a 26.82% reduction in the procurement volume of parenteral administered restricted antibiotics, a 33.64% reduction in the cost of parenteral administered restricted antibiotics, and a 27.84% reduction in the procurement cost of non-restricted oral antibiotics. This is understandable because the administrative rules set up a very strong control over the use of restricted antibiotics (details in Box 1). Although there is a paucity in the literature documenting the effectiveness of restrictive measures on prescribing practices [15], a recent study shows that a financial penalty for violating the existing guidelines can decrease subsequent antibiotic prescriptions and associated costs [33]. There was no change in the price of medicines over the study period. Medicines for primary care facilities were procured through a provincial tendering system. The price of the procured medicines was fixed until the next round of the tendering process. Over the study period, there was no new round of tendering. Therefore, the decline in cost reflects a reduction in the volume of prescriptions, not a reduction in price.

The cost-saving effect of the restrictive interventions on the use of antibiotics in primary care settings should not be interpreted as an effect on the entire health delivery system for several reasons. First, the restrictive intervention may encourage more referrals from primary care workers, increasing the cost associated with doctors’ time. Second, the restrictive intervention involves additional administrative costs. Third, it is also likely that some patients may bypass primary care and seek more expensive care from hospitals. In China, primary care is delivered in both primary care facilities and hospitals, and patients enjoy the freedom to choose their preferred primary care providers [34]. In 2011, China established a medication review system for antibiotic prescriptions, where a medication review team involving physicians and pharmacists is required to provide advice on the rational use of antibiotics [35]. But there is a lack of mechanisms for action. The auditing and penalty strategies were supposed to serve as an instrument for the medication review team to introduce actions. No additional investment is required.

Restrictive intervention strategies may have the potential to complement persuasive intervention strategies. A few studies reported the limited effects of guidelines on antibiotic prescribing practices. For instance, a national guideline recommended no initial antibiotic therapy on acute otitis media and adult sinusitis. But the rate of encounters at which no antibiotics was prescribed for these clinical conditions had not changed since the publication of the guideline [12, 13, 36]. However, an audit and feedback plus the distribution of a pocket version of the guidelines increased the prescribing compliance in a Norwegian hospital in terms of the right choice of empirical antibiotics, appropriate treatment duration, and decreased use of high-dose benzyl penicillin [37]. Public reporting may also help enhance the effects of practice guidelines, albeit in a small effect size [38, 39].

The intervention strategies tested in this study comprise multifaceted measures, including prescribing guidelines, audit and feedback, administrative rules and penalties, The multifaceted approach is particularly important in a health system where prescribers have varied qualifications and financial incentives are not always well aligned with the quality of care [33], as is often the case in low and middle income countries.

No significant impact on the overall cost or volume of antibiotics was detected in this study, although the results were numerically lower in all cases except the change in volume of non-restricted orals and the trend in the volume of restricted orals. Clearly, the overconsumption of antibiotics remains a significant challenge in China, despite the enormous efforts made by the government. Empirical evidence shows that economic motivation plays a crucial role in physicians’ prescribing practices [40]. The Chinese government has tried to decouple the link between the income of physicians and the sale of prescribed medicines through its EML and zero markup policies. However, it has resulted in a substantial loss of revenue for primary care facilities [41, 42] due to a shortage of government subsidies. These facilities have to turn their attention to other avenues to compensate for the loss, including user charges for the parenteral administration of medicines [38]. The regional centralized procurement arrangement does not forfeit the autonomy of health facilities to decide what and how much they can spend on medicines [43, 44]. In such a system context, persuasive measures alone barely have any significant impact on prescribing practices. The Antibacterial Use in Clinical Practice (2004) was proved to be fragmented and incomplete and has made only limited progress in containing antibiotic resistance [3, 45]. A coordinated systems approach may further tackle the issue of the irrational use of antibiotics.

The findings of this study have significant policy implications: administrative restrictive measures have the potential to lower antibiotic consumptions. However, it is important to note that the effect size of the tested intervention is rather small and antibiotic consumption remains high, especially parenteral administered antibiotics. Overall, antibiotics accounted for 33.65% of the total cost of procured medicines for primary care institutions in Hubei, much higher than the average level (11–18%) across all healthcare settings in Shanghai [46] or children’s hospitals (17.1%) in the US [47]. Antibiotics administered through the parenteral route still comprise over half of DDDs of antibiotics, contributing to almost 80% of the total cost of antibiotics. Since the 2009 health system reform, primary care institutions in China are no longer able to make a profit from dispensing medicines. However, they are allowed to charge a fee for services (injections) and consumables (syringes). This has added complications to the efforts to curb the overuse of injections. There is a need to organize a coordinated systems approach to tackle this issue.

This study has several strengths. The SMPA dataset includes the procurement records for almost all the primary care institutions in Hubei, covering all essential medicines. The institutionalization of longitudinal data reporting and a control group in this study avoids much of the bias of sampling. The use of cardiovascular medicines was unlikely to involve any significant changes over the study period, because primary care facilities were only allowed to dispense medicines listed in the EML which remained unchanged over the study period and the age structure and disease pattern of the population had limited, if any, changes in such a small time window (31 months). We also used more sophisticated methods, “interrupted time series”, which can make a more precise estimation of the policy impact compared with a simple pre-post comparison [48]. The sensitivity tests further enhanced the reliability of the results.

There are several limitations in this study. First, the data used in this study were drawn from procurement records, which do not directly reflect the actual use of medicines. We were unable to evaluate the appropriateness of antibiotic usage at the individual patient level. Second, prescriptions in private primary care facilities and the use of non-prescribed antibiotics (e.g. over-the-counter and leftover antibiotics at home) were excluded in this study. However, the impact of such exclusion is anticipated to be minimal. In 2012, primary care institutions received 68.51% of total outpatient visits in Hubei province. The participating community/township health centers in this study covered 86.36% of all primary care institutions in Hubei. Patients who visit health facilities usually fill their prescriptions at the same facility [44, 49]. We used multiple models and multiple tests to ensure the robustness of the study findings. However, some significant differences might arise due to chance where multiple tests were performed.

## Conclusions

Administrative restrictive regulations on antibiotic prescriptions are effective in reducing both the cost and volume of parenteral administered restricted antibiotics (26.82% reduction in volume and 33.64% reduction in cost). However, the restrictive interventions have failed to have a significant impact on overall cost and volume of antibiotic procurement. It is also important to note that costs may shift to other professionals and providers as a result of restrictions on primary care. Antibiotic usage remains high in Hubei, China, especially parenteral administered antibiotics. A coordinated systems approach is needed to further tackle the issue of the irrational use of antibiotics.

## Abbreviations

LMIC: low and middle income countries
AMR: antimicrobial resistance
EML: Essential Medicines List
ATC: Anatomical Therapeutic Chemical
HMPA: Hubei Medical Procurement Administrative Agency
DDD: defined daily dose
WHO: World Health Organization
DPP: defined daily dose equivalence per package
